# Strong negative self regulation of Prokaryotic transcription factors increases the intrinsic noise of protein expression

**DOI:** 10.1186/1752-0509-2-6

**Published:** 2008-01-18

**Authors:** Dov J Stekel, Dafyd J Jenkins

**Affiliations:** 1 Centre for Systems Biology, School of Biosciences, University of Birmingham, Edgbaston B15 2TT, UK

## Abstract

**Background:**

Many prokaryotic transcription factors repress their own transcription. It is often asserted that such regulation enables a cell to homeostatically maintain protein abundance. We explore the role of negative self regulation of transcription in regulating the variability of protein abundance using a variety of stochastic modeling techniques.

**Results:**

We undertake a novel analysis of a classic model for negative self regulation. We demonstrate that, with standard approximations, protein variance relative to its mean should be independent of repressor strength in a physiological range. Consequently, in that range, the coefficient of variation would increase with repressor strength. However, stochastic computer simulations demonstrate that there is a greater increase in noise associated with strong repressors than predicted by theory. The discrepancies between the mathematical analysis and computer simulations arise because with strong repressors the approximation that leads to Michaelis-Menten-like hyperbolic repression terms ceases to be valid. Because we observe that strong negative feedback increases variability and so is unlikely to be a mechanism for noise control, we suggest instead that negative feedback is evolutionarily favoured because it allows the cell to minimize mRNA usage. To test this, we used *in silico *evolution to demonstrate that while negative feedback can achieve only a modest improvement in protein noise reduction compared with the unregulated system, it can achieve good improvement in protein response times and very substantial improvement in reducing mRNA levels.

**Conclusion:**

Strong negative self regulation of transcription may not always be a mechanism for homeostatic control of protein abundance, but instead might be evolutionarily favoured as a mechanism to limit the use of mRNA. The use of hyperbolic terms derived from quasi-steady-state approximation should also be avoided in the analysis of stochastic models with strong repressors.

## Background

Recent innovations in synthetic biology and real-time imaging have revealed that the abundance of individual proteins in single cells is subject to significant variation, both between cells, and temporal variation within single cells, typically measured in unicellular organisms such as *Escherichia coli *and *Saccharomyces cerevisiae *[1-5]. Such variability is expected and confirmed by mathematical models of protein production, which have demonstrated that protein abundance is subject to random fluctuations resulting from intrinsic and extrinsic noise associated with transcription, translation and mRNA and protein degradation [6-9].

It is important for cells to control the abundance of individual proteins. There are many strategies that cells can employ, including the control of transcription, translation and the degradation of mRNA and protein. One important strategy is the employment of transcription factors – proteins that either positively or negatively regulate transcription.

Many transcription factors have the capacity to control their own transcription, usually in a negative fashion. This is particularly the case in prokaryotes; for example in the best studied organism *E. coli *K12, 79 of the 146 transcription regulators listed on Ecocyc [10] control their own expression – the majority of these are negative feedback. Typically, these regulators are associated with operons containing other proteins, sometimes on the same strand (e.g. *E. coli *NikR), but more commonly divergently transcribed (e.g. the *E. coli *proteins CynR, AraC, SoxR, MelR and many others). Therefore this mechanism is not just controlling the transcription factor itself, but also a gamut of associated proteins, typically working together in the same functional system.

Because so many prokaryotic genes are controlled by negative self-regulating transcription factors, it would appear that such that self-regulation is favoured by evolution. This begs the question of why it is favoured: what is the functional role of negative self-regulation in transcription systems?

The most commonly quoted answer comes from engineering principles. Negative feedback as a mechanism of control is as common in engineering as it is in biology, and therefore it has been natural to conclude that it must be playing similar roles. A simple engineering example is the thermostat, which uses negative feedback to maintain a desired temperature in a room. If the temperature is too cold, heating is switched on; if it is too warm, heating is switched off. In biology, there are many macro-physiological examples of homeostatic control using negative feedback, for example the control of blood sugar level using insulin and glucagon.

This would lead to the view that negative self regulation enables homeostatic control; the cell can use negative self regulation to maintain protein expression at a desired level [11-13]. Other authors have also demonstrated that negative feedback can shift the noise spectrum from low to high frequencies [14-16]. Control of noise would appear to be particularly important in the light of the stochasticity of protein expression observed in real cells. Because protein expression is subject to intrinsic and extrinsic noise, it is even more important to provide homeostatic control of that expression.

A very different explanation is suggested by Rosenfeld *et al*. [17]. They analyze ODE models of negative self regulation and conclude that such systems are able to substantially reduce the response time of protein production in the event of environmental change.

However, there are fundamental differences between transcription systems and the control of temperature or of relatively abundant metabolites (e.g. glucose). The number of molecules in transcription systems is necessarily small. Even if a transcription factor itself is relatively abundant, one of the most important molecules, the DNA molecule, is present in only in a small number of copies, depending on cell cycle and the proximity of the associated gene to the origin of replication. In mathematical terms, models based on ordinary differential equations (ODEs) can often be used to describe the average behaviour of a large population of cells. However, evolution acts on individual cells, and differential equation models of transcription regulation are not valid at the individual cell level. It is impossible to understand the functional role of transcription motifs without evolutionary context, and so it is vital to explore mathematical models that are valid at the single-cell level.

Therefore we have carried out a theoretical investigation of the role of negative transcription regulation on the variability of protein expression using stochastic models. The models that we analyze are similar to those studied by Thattai and van Oudenaarden [7] and Simpson *et al*. [14].

### Models of Negative Self Regulation

Thattai and van Oudenaarden analyzed a stochastic model for a negatively self-regulated gene. In this model, there are six processes: protein binding to DNA, protein-DNA complex dissociation, mRNA production, mRNA degradation, protein production and protein degradation. Of course, each of these processes themselves consists of many sub-processes, for example mRNA production includes the binding of RNA polymerase, initiation, multiple elongation steps and termination. Some authors have built more complex mathematical models that explicitly include these sub-processes [18]. The model also does not include a number of important cellular processes, notably DNA and cell replication.

The model is constructed by considering each of the possible transitions that can take place. This defines a continuous time Markov Chain with the following transitions:

• *D *↦ *D *- 1; *P *↦ *P *- 1 at rate *k*_*on*_*DP*

• *D *↦ *D *+ 1; *P *↦ *P *+ 1 at rate *k*_*off *_(1 *- D*)

• *M *↦ *M *+ 1 at rate *k*_*m*_*D*

• *M *↦ *M *- 1 at rate *γ*_*m*_*M*

• *P *↦ *P *+ 1 at rate *k*_*p*_*M*

• *P *↦ *P *- 1 at rate *γ*_*p*_*P*

*D*, *M *and *P *represent the numbers of DNA, mRNA and protein molecules respectively. *k*_*on *_represents the rate of protein binding to DNA, *k*_*off *_is the dissociation rate of the DNA-protein complex, *k*_*m *_is the rate of transcription, *k*_*p *_the rate of translation, *γ*_*m *_the rate of degradation of mRNA and *γ*_*p *_the rate of degradation of protein. (Here, we have slightly changed the notation of Thattai and Van-Oudenaarden). Thattai and Van Oudenaarden derive an elaborate term for the fano factor, also known as noise strength, which is defined as the ratio of protein variance to protein mean, in two steps. First, they make the commonly-used quasi-steady-state (QSS) approximation that the rate of binding and dissociation of the transcription factor to the DNA is faster than the dynamics of the mRNA and protein production. This leads to a simpler system in which the rate of transcription is given by a Michaelis-Menten-like hyperbolic term:

• *M *↦ *M *+ 1 at rate



• *M *↦ *M *- 1 at rate *γ*_*m*_*M*

• *P *↦ *P *+ 1 at rate *k*_*p*_*M*

• *P *↦ *P *- 1 at rate *γ*_*p*_*P*

*k*_*d *_is the strength of the transcription factor binding site and is defined as *k*_*off*_/*k*_*on*_. (We have written these equations with no Hill coefficient, although it is possible to include such a coefficient into the model at this stage).

The second step is to use a Taylor series to linearize the model about the protein steady state. It is then possible to derive the variance for the linearized model using moment equations. Since the model is now linear, no moment closure techniques are required and an exact solution can be found.

However, somewhat surprisingly, Thattai and van Oudenaarden have presented the results of their model only for weak repressors with range of *k*_*d *_between 100 *nM *and weaker (their Figure 3b). In contrast, real self-regulating repressors typically have much stronger values of *k*_*d*_, in the range 0.01 *nM *to 100 *nM *. Examples of strong negative self-regulators include *E. coli *NikR, with *k*_*d *_of 0.015 *nM *[19], and *E. coli *PurR, with *k*_*d *_of 0.1 *nM *[20]. Other self-regulating transcription factors in the 1 *nM *to 100 *nM *range include *E. coli *ChbR with *k*_*d *_of 1 *nM *[21]; KorB from the RK2 plasmid with *k*_*d *_of 9.3 *nM *[22]; and *E. coli *Lrp with *k*_*d *_of 35 *nM *[23]. Interactions weaker than 100 *nM *are typically regarded as non-specific. Therefore it is difficult to draw conclusions about realistic systems from their presented results.

Simpson *et al*. use a Langevin approach to derive a frequency-dependent analysis of the same system. They make the same QSS assumption as Thattai and Van Oudenaarden to introduce a hyperbolic term for the repression and also linearize the system about its steady state. They derive a simpler expression for the fano factor and demonstrate that the fano factor for the negatively regulated system is equal to the fano factor of the unregulated system divided by 1 + *T*(0), where *T *is the loop transmission which measures the level of resistance of the system to changes in protein level. Importantly, they also demonstrate that the frequency of noise is shifted from lower to higher frequencies in the presence of negative regulation.

### Approaches Taken in This Work

We analyze this system using three different approaches that complement and extend these important contributions. First, we analyze the model using mathematical approaches similar to those above. However, in contrast with Thattai and Van Oudenaarden, we examine the model for physiologically realistic values of *k*_*d*_. By demonstrating that the system has two distinct behaviours in different parameter regions, and observing that only one of these parameter ranges is relevant for physically stable proteins with realistic values of *k*_*d*_, we are able to employ stronger approximations and derive a very simple expression for protein variance that we discuss in the light of the results of Thattai and Van Oudenaarden and Simpson *et al*. Second, we run stochastic simulations with realistic parameters and demonstrate that the results of mathematical analyzes such as ours or those of other authors are only good when the dynamics of DNA binding and dissociation are fast relative to changes in protein abundance. This allows a QSS approximation to be made that leads to the appearance of a hyperbolic, Michaelis-Menten or Monod type term for transcription of gene expression seen in the majority of models [7,14,17]. However, when the promoter dynamics are slower, as must be the case for stronger negative repression, as the DNA-protein complex is more stable, we demonstrate that these results cease to be valid, and we use computer simulations to establish the behaviour of the system – which is qualitatively and quantitatively different from systems with weaker negative regulation.

Third, we make use of *in silico *evolution as an approach to understanding the behaviours of the models. Real biological systems are the result of millions of years of evolution – an experiment that is impossible to repeat during a human life-span. Using *in silico *evolution, we can apply the principles of variation and natural selection to a mathematical model, and evolve "organisms" with parameters that are particularly good at solving a set task (e.g. to minimize the noise of protein expression). This allows us to study systems in an evolutionary context, and to perform experiments in evolution that are not feasible with wet biology. We use this approach to compare the effectiveness of unregulated and negatively self regulated systems at controlling protein noise, minimizing protein response time and minimizing mRNA usage. We conclude that the role of negative self regulation in transcription networks may not always be the homeostatic control of protein, especially if the regulation is strong. We put forward a new hypothesis that it may be a strategy for energy minimization that allows the production of protein with minimum access to DNA and use of ribonucleotides. We propose a set of experiments that could be carried out to verify or falsify our results.

## Results and Discussion

### Mathematical Models: No Regulation

The means and variances for the model in which there is no regulation provide a useful baseline to compare with the models with negative self regulation. In this model, *k*_*on *_is set to zero so that only the transitions for mRNA and protein production and degradation appear. All parameters in these models are expressed in terms of molecules per cell. To convert parameters to molar units, it is necessary to multiply parameters by 10^-9^, which corresponds to a spherical cell of diameter 1. 5 *μm*.

Using Equation 11 (see methods), it is possible to derive differential equations for both the mean and variances of the mRNA and protein molecules (see Additional file 1), denoted by $\hat{M}$, $\hat{P}$ (means) and var(*M*) and var(*P*) (variances), given by:

$$\hat{M}=\frac{k_{m}}{\gamma_{m}}$$

$$\mathrm{var}(M)=\frac{k_{m}}{\gamma_{m}}$$

$$\hat{P}=\frac{k_{m}k_{p}}{\gamma_{m}\gamma_{p}}$$

$$\mathrm{var}(P)=\hat{P}\left(1+\frac{k_{p}}{\gamma_{m}+\gamma_{p}}\right)$$

There are four important consequences of this model: (i) The number of mRNA molecules follows a Poisson distribution – this result is already established in classical mathematics as the model for the number of mRNA molecules is equivalent to the standard birth-death process. (ii) The variance of the number of protein molecules is proportional to the mean, so that the ratio of variance to mean (the fano-factor) is a natural measure of intrinsic noise alongside the ratio of standard deviation to mean (coefficient of variation). (iii) The variance of the number of protein molecules is at best the variance associated with Poisson noise (when *k*_*p *_≪ *γ*_*m *_+ *γ*_*p*_); for larger values of *k*_*p*_, the variability is greater than Poisson variability. (iv) For biologically realistic parameters for transcription, translation and mRNA and protein degradation, variance divided by mean would range between 8 and 500 molecules per cell.

### Mathematical Models: Negative Self Regulation

The first part of the analysis proceeds in the same way as Thattai and Van Oudenaarden by making the QSS approximation and introducing a hyperbolic term for the production of mRNA. We now observe that the reduced model has two distinct behaviours depending on whether the dynamics are occurring in a saturated or non-saturated state (for full derivation, see Additional file 1). First, if ${\hat{P}}_{u}$ the steady state mean level of protein in the system without regulation, equal to *k*_*m*_*k*_*p*_/*γ*_*m*_*γ*_*p*_, is less than *k*_*d*_/4, then the model is approximately the same is the unregulated model. This is the non-saturated state. Insufficient protein is produced for the effect of the repression to be relevant. The more interesting case is when ${\hat{P}}_{u}$ > *k*_*d*_/4. This is the saturated case; it is also the more physiological case for any stable protein because the protein will continue to be made until it switches itself off – which can only be when the concentration exceeds the *k*_*d*_. In this case, the rate of transcription is approximately *k*_*m*_*k*_*d*_/*P *. The steady state mean values for ${\hat{M}}_{n}$ and ${\hat{P}}_{n}$ are given by:

$${\hat{M}}_{n}=\sqrt{\frac{k_{m}\gamma_{p}k_{d}}{\gamma_{m}k_{p}}}$$

$${\hat{P}}_{n}=\sqrt{\frac{k_{m}k_{p}k_{d}}{\gamma_{m}\gamma_{p}}}$$

In line with other authors, we derive an expression for the variance of the model that has been linearized about its steady state. The linearization is achieved by making the substitution

$$P={\hat{P}}_{n}(1+(\frac{P}{{\hat{P}}_{n}}-1))$$

By using the Taylor expansion for (1 + *x*)^-1 ^it then is possible to derive a linear model that approximates the nonlinear model and for which it is possible to derive analytic terms for moments (see Additional file 1 for details of the mathematics). The protein variance of the linearized model is given by:

$$\mathrm{var}(P)={\hat{P}}_{n}\left(1+\frac{k_{p}-\gamma_{m}}{2(\gamma_{m}+\gamma_{p})}\right)$$

Although the Poisson-like nature of the noise makes the fano coefficient a natural description of variability, a more standard measure is the dimensionless coefficient of variation equal to standard deviation divided by mean. By combining Equations 6 and 8 it can be seen that the coefficient of variation is proportional to $1/\sqrt{k_{d}4}$

$$\text{cv}(P)=\sqrt{\frac{\gamma_{m}\gamma_{p}}{k_{m}k_{p}k_{d}}4}\sqrt{1+\frac{k_{p}-\gamma_{m}}{2(\gamma_{m}+\gamma_{p})}}$$

This means that the coefficient of variation actually increases as the strength of the repressor increases. An alternative controlled comparison is to vary *k*_*d *_while retaining the same protein expression. One natural way of doing this is to vary the RNA polymerase promoter strength (implicitly included in *k*_*m*_) in concert with *k*_*d *_so that their product is constant and consequently protein level remains constant as repression increases. This can be thought of in terms of the cell employing different strategies to control a protein to a set level, ranging from a weak promoter and weak feedback through to a strong promoter with strong feedback. In that case, it can be seen from Equation 9 that the coefficient of variation should also be independent of repressor strength.

The repressor system is able to show some improvement in repression when cooperativity is included in the model. With a Hill coefficient of *n*, the protein variance equation becomes:

$$\mathrm{var}(P)={\hat{P}}_{n}\left(1+\frac{k_{p}-n\gamma_{m}}{\left(n+1)(\gamma_{m}+\gamma_{p}\right)}\right)$$

However, the result that the fano factor is independent of repressor strength, and the equivalent result for the coefficient of variation, still hold in this system.

**Figure 1:**
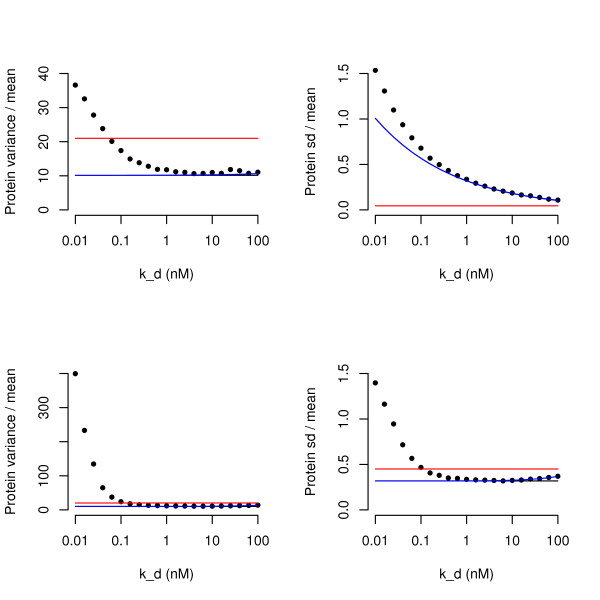
**Dependence of Protein Noise on *k*_*d*_**. Dependence of fano factor (variance of number of protein molecules per cell divided by mean number of protein molecules per cell) and coefficient of variation (standard deviation divided by mean) on *k*_*d *_of the DNA binding site, for physiological values of *k*_*d *_ranging between 0.01 *nM *and 100 *nM *. In all panels, *k*_*p *_= 0.1*s*^-1^, *γ*_*m *_= 5 × 10^-3^*s*^-1 ^and *γ*_*p *_= 2 × 10^-4^*s*^-1^. In the top two panels (a) and (b), *k*_*d *_is varied, and the model is controlled by holding all other parameters fixed. In the bottom two panels (c) and (d), *k*_*d *_is varied, and the model is controlled to keep a constant protein abundance of 100 molecules per cell by also varying *k*_*m*_. In the left-hand panels (a) and (c), the fano factor is plotted as a noise measure; in the right-hand panels (b) and (d), the coefficient of variation is plotted. In (a) and (b) *k*_*m *_= 0.1*s*^-1^. In (c) and (d), *k*_*m *_is varied along with *k*_*d *_so that mean protein abundance is held constant. Each of the data points is the measure of noise from a stochastic simulation of the model. The black lines show the respective noise measure as derived by our mathematical analysis; the blue lines, where distinguishable from the black lines, show the noise measure as derived by Thattai and van Oudenaarden; the red lines show the noise measure for the equivalent unregulated model. In all panels it can be seen that (i) our noise measures are very close to the expression derived by Thattai and Van Oudenaarden; (ii) the simulations match the noise level for weak values of *k*_*d *_greater than 1 *nM *; (iii) for strong values of *k*_*d *_less than 1 *nM*, the level of noise increases with repressor strength, and is very much greater than predicted by the linearized QSS model. (a), (c) and (d) all show that the noise level is predicted to be lower in the regulated system than the equivalent unregulated system. However, the stochastic simulations demonstrate that for strong values of *k*_*d*_, the noise of the regulated system can be greater than that of the unregulated system. In (b), the red line would appear to indicate that the unregulated system is consistently less noisy than the regulated system. However, in this panel, because all parameters are held constant, the protein abundance is much higher than in the unregulated system than the regulated system, and because of the Poisson-like nature of the noise (variance proportional to mean), the coefficient of variation is necessarily lower. In (d) it can be seen that when the unregulated system is adjusted so that protein levels are the same, a consistent pattern of behaviour is observed.

This result might appear to be different from that of Thattai and van Oudenaarden [7], but in fact there is no conflict between these results. The mathematical analysis is representative of a realistic parameter range in which it is possible to make stronger approximations than Thattai and van Oudenaarden and thus to derive a formula that is simpler and clearer. Thus we have demonstrated that the formula of Thattai and van Oudenaarden has the asymptotic property that variability is independent of *k*_*d *_for strong repressors. When the two formulae are plotted against alongside other in this range, they give almost identical results (Figure 1).

The result is also consistent with, although slightly different from, the result of Simpson *et al*. [14]. Where the protein is fully saturated, their loop transmission term *T *would be equal to 0, and so the fano factor would be independent of the *k*_*d *_and in fact equal to the fano factor for the unregulated system. Our analysis goes a step further and shows that even for a range where the negative regulation is effective, the noise should be independent of *k*_*d*_.

### Computer Simulations

**Figure 2:**
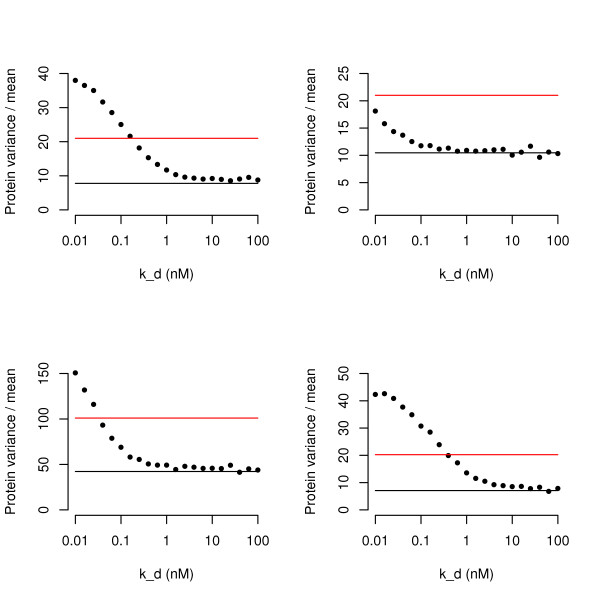
**Dependence of *k*_*d *_Noise Response on Other Parameters**. The qualitative nature of our results are independent of choice of parameter, although the quantitative measures do change. All panels show the fano factor (variance divided by mean) for varying values of *k*_*d *_holding all other parameters constant (a very similar pattern would be seen using coefficient of variation and/or controlling for constant protein expression). Importantly, the mathematical formulae only fit the simulations for weak *k*_*d*_, and the noise increases for strong repressors. (a) A less stable protein with *γ*_*p *_= 2 × 10^-3^*s*^-1 ^exhibits similar behaviour; all other parameters are as in Figure 1(a). (b) A very stable protein with *γ*_*p *_= 2 × 10^-5^*s*^-1 ^exhibits the same behaviour, except that with these parameters, the increase in fano factor never matches the noise of the unregulated system. (c) A more stable mRNA with *γ*_*m *_= 10^-3^*s*^-1^. (d) A cooperatively binding protein with Hill coefficient of 2 also shows the same pattern, but this time the noise is greater than the unregulated system for weaker repressors than in the non-cooperative case.

Because of the many approximations needed to obtain a closed form mathematical estimate for the variance of the protein level, we also ran computer simulations for the dependence on variance on the promoter strength and other parameters. Figure 1 shows how the fano factor and the coefficient of variation depend on repressor strength for different realistic values of *k*_*d*_, using a set of realistic parameters for mRNA and protein stability (variations in these parameters are explored in Figure 2). The model is controlled both by holding all other parameters constant, or by adjusting the value of *k*_*m *_so that the expected protein level is constant.

The figure demonstrates a number of important points. First, for weaker repressors, with *k*_*d *_> 1 *nM*, the expressions that we derive is consistent with both the simulated data, in that the fano factor and the coefficient of variation are approximately independent of *k*_*d *_when protein level is controlled. Second, for all parameter ranges, there is very close fit between our simple expressions for protein noise and the more complicated expression of Thattai and Van Oudenaarden, with the exception of very weak repressors in Figure 1(d) where their expression fits the data slightly better. Most importantly, however, is that as repressor strength increases, with *k*_*d *_< 1 *nM*, both the fano factor and the coefficient of variation increase to much higher levels than those predicted by linearized QSS. For the parameters used in Figure 1, when *k*_*d *_< 0.1 *nM*, both the fano factor and coefficient of variation have risen to levels greater than those of the equivalent unregulated model.

In Figure 2 we demonstrate that the increase in noise for strong repressors is qualitatively (although not quantitatively) independent of choice of parameters, by showing graphs of fano factor against *k*_*d *_for less stable protein (Fig 2a), more stable protein (Fig 2b), more stable mRNA (Fig 2c) and the inclusion of a cooperativity (Hill) coefficient of 2 (Fig 2d). Although the graphs show the same qualitative behaviour, one important quantitative difference is the value of *k*_*d *_for which the repressed system becomes more noisy than the unregulated system. With a very stable protein (13 hours) the negative regulator is always less noisy for realistic values of *k*_*d*_; however, this parameter is difficult to interpret for exponentially growing cells in which protein turn-over would be limited by the dilution rate and so is likely to be of relevance only in stationary phase. Interestingly, when cooperatively of protein binding is included, the protein abundance is noisier for weaker values of *k*_*d *_than without cooperativity. Very similar behaviours are observed for other realistic parameter values (see Additional file 1).

**Figure 3:**
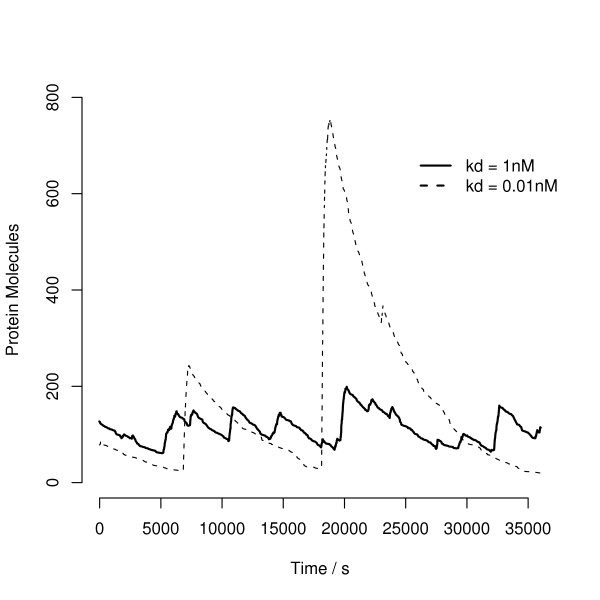
**Bursty Protein Production**. Two simulations contrasting the behaviour of protein abundance for a moderate repressor with *k*_*d*_of 1 *nM *(solid line) and a strong repressor with *k*_*d *_of 0.01 *nM *(dashed line). For the 1 *nM *repressor, all other parameters are as in Figure 1(a). For the strong repressor, *k*_*m *_has been adjusted so that the both models have an average protein abundance of 100 molecules per cell – this allows both simulations to be plotted on the same axes so that the noise can be easily compared. In both cases, protein is produced in bursts. With the moderate repressor, the bursts are short and protein level is being continuously adjusted about the mean. With a strong repressor (low *k*_*d*_), the bursts are large and coincident with small number of times in this simulation that mRNA is synthesized. This is the source of the additional variability over and above the linearized system. It is also important to observe that the variability in protein abundance – at least for a stable protein – is slow relative to the cell cycle time. This means that extrinsic noise due to DNA and cell replication are likely to contribute very significantly to strongly auto-repressing transcription factors.

Figure 3 shows a time course for part of two simulations, one with an average *k*_*d *_of 1 *nM *and the other with a strong *k*_*d *_of 0.01 *nM *. In order to compare the two behaviours on the same axes, we have controlled the two simulations by adjusting *k*_*m *_so that the expected protein level is the same. In both cases, protein production is in bursts, coincident with the synthesis of mRNA when the repressor dissociates from the DNA. But while with average *k*_*d*_, protein abundance is adjusted reasonably quickly, when the *k*_*d *_is strong, the bursts are slow and irregular. With these particular parameters, the bursts are quite slow relative to the cell cycle time, and therefore it is likely that DNA replication and cell division will interact significantly with protein synthesis and contribute extrinsic noise. These results also appear to contrast with those of Simpson *et al*. in that strong negative repression appears to shift the noise to lower frequencies rather than higher ones.

### *In Silico *Evolution

**Figure 4:**
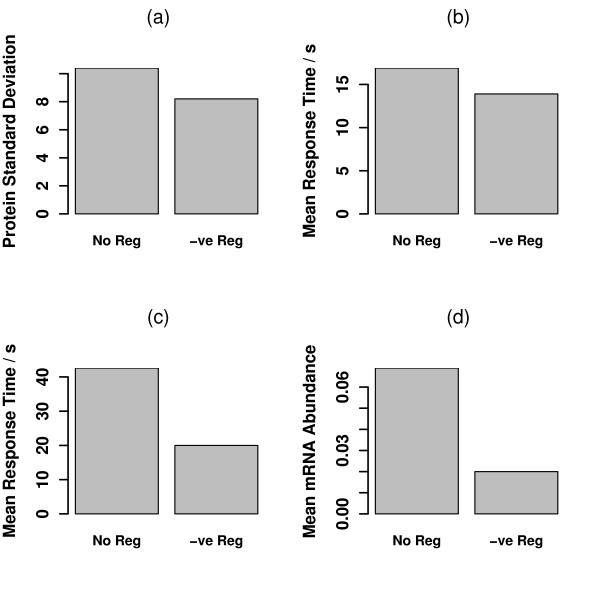
**In Silico Evolution**. Simulations of the bestmodels derived from *in silico *evolution of the systemwithout regulation and the system with negative feedback. (a) Evolution to minimize protein standard deviation. The regulated system can achieve about 21% improvement over the unregulated system, with the standard deviation reduced from 10.4 to 8.2 for a protein abundance of 100 molecules per cell. The evolved parameters for the unregulated system are: *k*_*m *_= 1.0*s*^-1^; *k*_*p *_= 0.00387*s*^-1^; *γ*_*m *_= 0.0664*s*^-1^; *γ*_*p *_= 0.000572*s*^-1^. The evolved parameters for the negatively regulated system are: *k*_*off *_= 0.256*s*^-1^; *k*_*m *_= 0.835*s*^-1^; *k*_*p *_= 0.294*s*^-1 ^*γ*_*m *_= 1.783*s*^-1^; *γ*_*p *_= 000282*s*^-1^. (b) Evolution to minimize first passage time to 50% of mean protein abundance. The regulated system only achieves an improvement of 18% over the unregulated system with the rise time (of 20 repeats of the best model) reduced from 16.9*s *to 13.9*s*. The evolved parameters for the unregulated system are: *k*_*m *_= 1.0*s*^-1^; *k*_*p *_= 1.0*s*^-1^; *γ*_*m *_= 0.0591*s*^-1^; *γ*_*p *_= 0.163*s*^-1^. The evolved parameters for the negatively regulated system are: *k*_*off *_= 0.0664*s*^-1^; *k*_*m *_= 1.0*s*^-1^; *k*_*p *_= 1.0*s*^-1^; *γ*_*m *_= 0.0494*s*^-1^; *γ*_*p *_= 0.0146*s*^-1^. (c) Evolution to minimize first passage time to mean protein abundance. The regulated system achieves an improvement of 53% over the unregulated system with the rise time (of 20 repeats of the best model) reduced from 42.6*s *to 20.0*s*. The evolved parameters for the unregulated system are: *k*_*m *_= 0.903*s*^-1^; *k*_*p *_= 1.0*s*^-1^; *γ*_*m *_= 0.101*s*^-1^; *γ*_*p *_= 0.0916*s*^-1^. The evolved parameters for the negatively regulated system are: *k*_*off *_= 0.000648*s*^-1^; *k*_*m *_= 1.0*s*^-1^; *k*_*p *_= 1.0*s*^-1^; *γ*_*m *_= 0.00139*s*^-1^; *γ*_*p *_= 0.0183*s*^-1^. (d) Evolution to minimize mRNA usage. The regulated system is able to achieve a 71% improvement over the unregulated system, reducing mean mRNA levels from 0.069 to 0.020 molecules per cell to achieve a protein abundance of 100 molecules per cell. The evolved parameters for the unregulated system are: *k*_*m *_= 0.000641*s*^-1^; *k*_*p *_= 0.468*s*^-1^; *γ*_*m *_= 0.0148*s*^-1^; *γ*_*p *_= 0.000200*s*^-1^. The evolved parameters for the negatively regulated system are: *k*_*off *_= 0.0942*s*^-1^; *k*_*m *_= 0.270*s*^-1^; *k*_*p *_= 1.0*s*^-1^; *γ*_*m *_= 1.168*s*^-1^; *γ*_*p *_= 0.000200*s*^-1^. Note that the systems that minimize noise and mRNA usage evolve stable proteins while the systems that minimize response times evolve more rapidly turned-over proteins.

Four different *in silico *evolution experiments were performed: the first to minimize the standard deviation of protein expression; the second to minimize the rise time to half the steady state of protein expression; the third to minimize the rise time to the steady state protein expression; and the fourth to minimize mRNA abundance. In each experiment, the model with no regulation was compared with the model with regulation. The results are shown in Figure 4.

In Figure 4a, it can be seen that the best evolved standard deviation of the unregulated system is 10.4 molecules per cell; the minimum standard deviation of the system with negative regulation is 8.2 molecules per cell. This represents a 21% decrease in standard deviation of protein expression. Observe that the Poisson noise level for 100 copies of the protein would be 10 molecules per cell; the unregulated system has evolved to match the Poisson noise, while the regulated system has evolved a noise level slightly below that. In Figure 4b it can be seen that the best evolved rise time of the unregulated system to half its steady state level is 16.9*s*; the best evolved rise time of the regulated system is 13.9*s*. This represents an 18% decrease in rise time for the self regulated system. In Figure 4c it can be seen that the best evolved rise time of the unregulated system to its steady state level is 42.6*s*; the best evolved rise time of the regulated system is 20.0*s*. This represents an 53% decrease in rise time for the self regulated system. In Figure 4d, it can be seen that the best evolved mRNA level of the unregulated system is a time average of 0.069 molecules per cell; the best evolved mRNA level of the system with negative regulation is 0.020 molecules per cell. This represents a 71% decrease in mRNA usage to 29% of the level of the unregulated system. Therefore there is a clear hierarchy of improvements of these three goals: modest improvements in the reduction of noise and rise-time to 50% steady-state level, good improvements in rise time to steady state level, and very substantial improvements in mRNA usage.

## Discussion

The number of molecules of a protein in a single cell varies in time, resulting from stochasticity in the processes of transcription, translation and degradation. This variability extends also to variability between individual cells in a population. This variation can be seen in experiments that track gene and protein expression in single cells [1,3] and in experiments that track protein expression in a population of cells [2]. Many transcription systems include genes that negatively regulate their own expression [10]. It has been proposed that the role of such negative regulation is to ensure homeostatic control of the protein products a view deriving from our understanding and use of negative feedback in engineering [11,12]. In this study, however, we demonstrate the reverse: strong negative control of gene expression results in increased variability.

We have carried out an investigation of how the level of variation depends on the rates of key processes: transcription, translation, degradation of mRNA and protein, and the binding and dissociation of the transcription factor to the DNA regulatory site. The analysis has been on three levels: mathematical models, computer simulations and *in silico *evolution.

Analytic solution for the linearized version of the negative feedback loop suggests that the fano factor should be independent of repressor strength and that the coefficient of variability should increase with repressor strength, or also be independent of repressor strength if the model is controlled for constant protein level. While these results are themselves surprising, they are in fact implicit in the results of other authors [7,14], and our derivation provides a simple mathematical formula to capture the behaviour in terms of other parameters.

More importantly, however, we show that these results are only applicable when the DNA-protein binding dynamics are fast relative to the dynamics of mRNA and protein production and degradation. Instead, computer simulations reveal that as repressor strength increases, so too does the noise. While the noise for weak repressors (with *k*_*d *_much bigger than 1 *nM*) approaches the asymptotic limit derived from the mathematical analysis, the fano factor and coefficient of variation of strong repressors (with *k*_*d *_much less than 1 *nM*) are very much higher than predicted by theory. For very strong repressors (with *k*_*d *_less than 0.1 *nM*), the noise can be greater than in an unregulated system. This is likely to be physiologically important, as many auto-regulating operators have *k*_*d*_s in this range, for example *E. coli *NikR, with *kd *of 0.015 *nM *[19], or *E. coli *PurR, with *k*_*d *_of 0.1 *nM *[20].

This result would appear to be in contrast with previous theoretical results [7], despite analyzing the same model. However, there are two important differences between the two analyses: first, that work investigated values of *k*_*d *_ranging from 100 *nM *and weaker (their Figure 3), which is weaker than many physiological repressors that operate in the range between 0.01 *nM *and 100 *nM*, and also outside the range in which the approximations used in the mathematical derivation cease to be valid. Furthermore, by observing the fact that for stable proteins the protein abundance will be in excess of the *k*_*d*_, we are able to derive a novel, clearer and simpler numerical expression for protein variability, and which gives essentially the same results.

The reason for this increase in noise is that when the repression is strong, protein molecules are produced in bursts, which result in highly variable protein levels [24,25]. These bursts are happening on a timescale related to the repressor strength that is slow relative to the dynamics of protein production and degradation. As a result, the standard QSS approximation, based on a separation of timescales between transcription factor binding events and protein production and degradation events, that leads to a hyperbolic term is not valid. Therefore the mathematical derivations that depend on it (including our own) cease to describe the behaviour of the model. This result is consistent with other situations in which stochastic models can behave differently from classical chemical kinetics, for example when stochastic switching can occur [26].

This also explains the apparent discrepancy between our results and those of Simpson *et al*.. They have shown, both with theory and experiments [15], that negative repression shifts the noise from low frequency to high frequency; our simulations have suggested that for some realistically strong values of *k*_*d*_, the noise increases and appears to be shifted to lower frequencies. However, the result of Simpson *et al*. is also derived using the QSS approximation that we have shown is valid for weaker repressors (*k*_*d *_> 1 *nM*) but not for stronger repressors (*k*_*d *_< 1 *nM*). Moreover, their experimental results were obtained using a TetR system that has a *kd *of 5.6 *nM *[27], which is in the range for which we would expect the QSS approximation to hold. Thus our findings are consistent with these results; we would predict that a similar experiment carried out with a stronger repressor (e.g. NikR or PurR) would give a quite different result. In fact, the behaviour we observe in models of strong repressors is consistent with the behaviour that the same authors derive for the open loop circuit that can be dominated by operator noise [28].

Another interesting point to emerge from this analysis is that for strong repressors, the timescale of fluctuations is slow relative to the rate of DNA or cell replication. The models that we have analyzed do not include terms for DNA or cell replication. Therefore it would appear likely that the interaction between protein production and replication may be quite strong. We would expect two consequences. Firstly, DNA replication may add significant extrinsic variability in these systems. Depending on position in the genome relative to the origin of replication, any given gene may be present in one, two or more copies at different times in the cell cycle [8]. This will influence both the mean and variance of protein expression, and a far more involved analysis would be necessary to evaluate the contributions of this effect to protein variability. Second, there is the capacity for epigenetic inheritance of protein abundance in these systems; this may be of benefit in some biological situations, for example stress responses.

A further interesting comparison is with the work of Kepler and Elston [6]. They apply a slow timescale approximation to a self-activating system and are able to derive an expression for the steady state probability density of protein abundance. This allows them to show that a self-activator can demonstrate bistable dynamics with a bimodal distribution of protein abundance. A similar approach might be successful for deriving an analytic expression for the noise in a negative self regulator that would be valid for physiologically strong repressors where the normal QSS cannot be used. However, the details would necessarily be different, as Kepler and Elston derive their result by considering the limit as average protein level tends to infinity – a procedure that may not be realistic when considering a repressor system. Therefore it appears that negative self regulation may not lead to better control of protein variability. And yet negative self regulation is favoured by evolution; if it is not playing an important role in regulating protein variability, then it must be performing a different function. One proposal is that negative regulation can speed up the time scale of the response of protein production to an environmental change [17]. We put forward a new proposal that strong negative regulation may provide a mechanism to produce protein for minimum use of resource – in particular mRNA usage and access to DNA. In the final part of our studies, we ran *in silico *evolution experiments to see how well different configurations of transcription control can adapt to different tasks. This allowed us to compare the effectiveness of negative self regulation at regulating noise, at mediating a rapid response time and to minimizing mRNA usage. We compared the negative feedback loop to the unregulated system. The negative feedback system only enjoyed modest improvements of about 20% over the unregulated system when minimizing the level of protein noise. Some improvement is to be expected as the system has five parameters as opposed to four, and this is supported by the analytic results. Better improvements of about 37% were seen in reducing response times, supporting the hypothesis of Rosenfeld *et al*. [17]. However, far more substantial improvements are seen when the systems are adapting to minimize the average mRNA levels, with 73% improvement with negative feedback as compared with the unregulated system.

Our hypothesis about minimizing mRNA usage makes sense in the context of plasmids, where many successful plasmids are of minimal burden to their hosts. It is common for the central regulators of plasmids to negatively regulate their own transcription, for example the KorB regulator in RK2 [29] or the *ω *regulator in pSM19035 [30]. Such mechanisms allow for a burst of gene expression on entry into a new cell to allow the plasmid replication, segregation and conjugation apparatus to become established, and then for the plasmid to switch off their genes and thus have minimal impact on the host once sufficient copy number is achieved. We propose that a similar idea holds for many constitutive bacterial genes too. Our hypothesis is also complementary with the rapid response hypothesis, in that rapid protein production following environmental change (e.g. cell division) is entirely consistent with subsequent shutting down of protein production. It is particularly interesting that using Rosenfeld *et al*.'s definition of the rise time to 50% of the steady state value produces very little improvement, while using a definition of rise time to the steady state value produces much better improvements. The reason for this is likely to derive from the fact that Rosenfeld *et al*. make use of ODE models, while we are using stochastic simulations. The ODE models cannot achieve the steady state value so it is necessary to use a fixed proportion of the steady state, and 50% is a natural proportion (analogous to *k*_*m*_). With stochastic models, however, the steady state is a mean about which the protein abundance varies, and so is always achieved. Thus it is quite reasonable to define the rise time as first passage time to the mean. It is possible that the improved performance of the negative self-regulator in reducing first passage time to mean value as opposed to half mean value is because the negative regulator can take advantage of the stochasticity of the system and allow an "overshoot" of protein production which can then be attenuated by negative regulation. This overshoot advantage is not seen with the half mean first passage time. It would appear that that the mean first passage time associated with stochastic models is a different property from rise time of the mean associated with ODE models. Moreover, Rosenfeld *et al*.'s study makes use of ODEs using the standard QSS and hyperbolic terms. Thus further investigation of rise times in stochastic, single-cell models is warranted. Furthermore, although strong negative repression might not be particularly effective at noise control, it is possible that cells might have evolved more elaborate mechanisms for noise control. These might include both negative and positive regulation, multiple transcription factors, or regulation at the mRNA or protein levels. An example of elaborate regulation that has been found to minimize noise is in the *E. coli *heat shock response [31]. It is likely that other mechanisms exist; *in silico *evolution techniques could prove valuable in identifying such potential mechanisms.

### Experimental Validation

The results of these analyses lend themselves quite naturally to experimental validation or falsification. The experiment would involve constructing a low-copy-number plasmid with a suitable negatively self-regulating transcription factor and its binding site, with very strong *k*_*d*_, controlling also the expression of GFP. A series of mutants would be made with mutations to the operator sequence so as to produce a series of weaker repression circuits. Bacteria lacking the aforementioned transcription factor can be transformed with this plasmid and grown. The mean and variance of GFP fluorescence in a population of cells would then be measured using flow cytometry. This procedure would be repeated for each of the mutant plasmid vectors. The DNA-protein *k*_*d *_values could also be measured for each operator sequence using biophysical techniques (accepting that such *in vitro *measurements may not always represent an accurate *in vivo k*_*d*_). From this data, it would be possible to plot protein variability against *k*_*d *_and thus determine whether or not variability really does increase within a physiological range of *k*_*d *_values.

## Conclusion

There are two important conclusions of these analyses. The first is that the standard quasi-steady-state approximation, in which it is assumed that the dynamics of protein-DNA binding are faster than the dynamics of mRNA and protein synthesis and degradation, and which gives rise to the hyperbolic Michaelis-Menten-like terms typically used in differential equation models of gene expression, is not realistic for a range of biologically important parameter values. New mathematical approximations will have to be derived in order to seek accurate closed-form equations for protein noise in such systems. Great care must be taken not to use hyperbolic terms in mathematical models of single cells, but instead to fully implement the protein-DNA interaction dynamics.

Second, strong negative self regulation can actually increase the noise of protein expression and is unlikely to be a mechanism for control of protein noise. Instead, we postulate that it is a mechanism to minimize the amount of mRNA needed to produce protein at a given level. There are two reasons why it might be advantageous for a cell to do this. First, it reduces the need to access the DNA molecule. A bacterium such as *E. coli *packs 4.5 MBp of DNA into a 1.5 *μm *cell: stretched out, the DNA molecule is about 1000 times the length of the cell, and it may be advantageous to minimize the extent to which the DNA needs to unfold and refold. The second explanation is that the energy required to manufacture mRNA. The synthesis of an mRNA molecule costs a cell approximately 1.5 times the energy of the correspondent protein molecule [32], and that does not take into account the energy invested in the nucleotides themselves. A variant of a gene that is able to produce the same amount of protein but using less mRNA may have significant evolutionary advantage over a version using more mRNA, both by saving on energy of mRNA synthesis, and by saving on ribonucleotide use. The negative feedback loop is a highly effective way of achieving this, and so is favoured by evolution.

Thus we see that negative self repression is not a single mechanism for homeostasis but is a mechanism that might be performing quite different functions depending upon the strength of the repressor. Weaker negative self-repressors can reduce noise and shift noise from low to high frequencies; stronger negative self-repressors increase noise, particularly at low frequencies. Negative self-repressors can speed up response times. And negative self-repressors, particularly strong self-repressors, allow protein production for minimal energy cost.

## Methods

### Derivation of Means and Variances

In order to derive equations for the means and variances of mRNA and protein expression, we make use of the equation:

$$\frac{d\langle f(x)\rangle }{dt}\langle \sum_{\text{events}}\text{rate of event}\times \text{change to }f(x)\text{ due to event}\rangle$$

In this equation, **x **represents a vector of variables and *f*(**x**) represents a function of **x**. It is straightforward to derive this equation from the Master Equation [33].

### Stochastic Simulations

Stochastic simulations of the systems studied have been carried out using the Gibson-Bruck algorithm [34], which has been implemented into our own Java-based simulator (source code is available from the authors on request). The simulations for Figure 1 were run with 1, 000, 000 protein production or degradation events. Parameters for these simulations were chosen for no particular gene but with a wide range of realistic parameters to explore the general behaviour of the model.

### *In Silico *Evolution

*In silico *evolution has been carried out as a genetic algorithm in a relatively standard way. An initial population of 25 individuals is generated; each individual has randomly chosen parameters. The no regulation model has four parameters and the negative regulation model has a fifth parameter, *k*_*off*_: *k*_*on *_is fixed at the physiological diffusion-limited level of 0.01 molecules per cell per second.

Initial parameters are chosen at random from the log (to base 10) of a normal distribution with mean of *-*2 and standard deviation 1 for all parameters (with appropriate units). At each round of the simulation, the best 25 individuals from the previous generation are selected (elitism); a further 25 individuals are generated by mutations, taking each individual and adding the log (to base 10) of normal noise with mean 0 and standard deviation 0.2 to each parameter (with appropriate units). The use of log normal mutations allows the model evolution to explore parameters at all orders of magnitude. A further 25 individuals are generated by recombination: two parents (elite or mutated) are chosen at random, and each parameter of the offspring is selected at random from one of the parents. The elite strategy was found to be superior to a Boltzmann random selection strategy (unpublished results) with faster convergence to similar limiting fitnesses.

In all individuals, we have constrained the rate of production of mRNA and the rate of production of protein to be within physiological realistic range. Thus mRNA and protein synthesis are each constrained to have maximum values of 1.0*s*^-1^. We have also constrained the rate of protein degradation to be no slower than a realistic cell cycle time; thus *γ*_*p *_can be no smaller than 0.0001995*s-*1 which corresponds to 83.5 minutes.

The fitness of each of the 75 individuals is determined on the basis of a simulations of the model with 100, 000 protein production and degradation events, and initial mRNA and protein levels of 0. The models were required to have a mean protein abundance of 100 molecules. The rise time is given as the first passage time either to half the required mean number of molecules, i.e. 50 molecules (to be consistent with the methodology of Rosenfeld et al.) or to the required mean number of molecules.

The fitness functions used are:

1. Deviance from protein mean + protein standard deviation

2. Deviance from protein mean + protein rise time

3. Deviance from protein mean + 10 times mRNA level

The scaling factor on the mRNA level is to ensure that the fitness functions weight their components comparably. In Figure 4 it can be seen that protein standard deviation is approximately 10 molecules per cell; rise times are approximately 20*s*; mRNA abundances are approximately 0.05 molecules per cell. Therefore in fact the fitness function is quite conservative for mRNA abundance and greater improvements could be seen with a higher weighting.

At each generation, the best 25 models are selected for the next generation. The genetic algorithms were run for 30 generations and each repeated 20 times. The best evolved models for each scenario were then simulated for 1, 000, 000 events to determine means and standard deviations. The rise times for the best models were evaluated as the average of 20 repeats.

## Authors' contributions

DS devised the work, carried out the mathematical research and wrote the paper. DJ implemented the stochastic simulation and evolutionary computation environments. Both authors have read and approved the final version of the manuscript.

## Supplementary Material

Additional file 1 Supplementary Information. Contains details of mathematical derivations and evolutionary simulations. Click here for file
