# Supplementary material for: Strong negative self regulation of Prokaryotic transcription factors increases the intrinsic noise of protein expression
Source: BMC Syst Biol. 2008 Jan 18;2:6. doi: 10.1186/1752-0509-2-6 (PMC2263017; doi:10.1186/1752-0509-2-6)
Supplement: Additional file 1 — Supplementary Information. Contains details of mathematical derivations and evolutionary simulations. [file 1752-0509-2-6-S1.pdf]

Strong negative self regulation of Prokaryotic transcription factors increases the intrinsic noise of protein expression: Supplementary Information

Dov J Stekel *
School of Biosciences
University of Birmingham
Birmingham B15 2TT
UK

Dafyd J Jenkins
School of Biosciences
University of Birmingham

January 10, 2008

Mean and variance of mRNA and protein abundance in unregulated system

The unregulated system is described by the set of reactions:

- $M \mapsto M+1$ at rate k_m
- $M \mapsto M-1$ at rate $\gamma_m M$
- $P \mapsto P+1$ at rate $k_p M$
- $P \mapsto P-1$ at rate $\gamma_p P$

Mean and variance of mRNA

The number of mRNA molecules is a standard birth-death process, a well-studied example in the theory of Markov Chains. As this is a time-reversible Markov Chain, an invariant distribution for the number of mRNA molecules can be derived using the time reversal equation (?). Let λ_i be the probability that there are i mRNA molecules and let $q_{i,j}$ be the transition rates associated with changing state from i molecules to j molecules. The time-reversal equation states that:

*to whom correspondence should be addressed

$$\lambda_i q_{i,j} = \lambda_j q_{j,i} \tag{1}$$

In this model, $q_{i,i+1} = k_m$ and $q_{i+1,i} = \gamma_m(i+1)$. No other transitions are possible. Therefore:

$$\frac{\lambda_{i+1}}{\lambda_i} = \frac{k_m}{\gamma_m(i+1)} \tag{2}$$

and so by induction,

$$\lambda_i = \frac{\lambda_0 (k_m/\gamma_m)^i}{i!} \tag{3}$$

We can determine λ_0 because

$$\sum_{i=0}^{\infty} \lambda_i = \lambda_0 \sum_{i=0}^{\infty} \frac{(k_m/\gamma_m)^i}{i!} = 1 \tag{4}$$

This is just an exponential function so $\lambda_0 = \exp(-k_m/\gamma_m)$ and so we see that

$$\lambda_i = \frac{e^{-k_m/\gamma_m}(k_m/\gamma_m)^i}{i!} \tag{5}$$

This is the probability distribution for a Poisson variable, so the number of mRNA molecules is distributed as a Poisson variable with parameter k_m/γ_m. Importantly, we deduce that both the mean and variance of the number of mRNA molecules are also k_m/γ_m.

Mean and Variance of Protein

The Markov Chain for the number of protein molecules is not time reversible, so this technique cannot be used to calculate the distribution of the number of protein molecules. Instead, we derive moment equations using the master equation and use the moment equations to derive the mean and variance of the invariant distribution (**?**). Because the system is linear, it is possible to derive exact solutions without resorting to moment-closure techniques. we make use of the equation:

$$\frac{d\langle f(\mathbf{x})\rangle}{dt} = \left\langle \sum_{\text{events}} \text{rate of event} \times \text{change to } f(\mathbf{x}) \text{ due to event} \right\rangle \tag{6}$$

In this equation, $\mathbf{x}$ represents a vector of variables and $f(\mathbf{x})$ represents a function of $\mathbf{x}$. For example, if $\mathbf{x}$ is a vector with two components M and P representing the number of mRNA molecules and protein molecules respectively, then the function for the variance of the number of protein molecules would be given by

$$f(M, P) = \langle P^2\rangle - \langle P\rangle^2 \tag{7}$$

and so the differential equation for the rate of change of variance of the mRNA $\text{var}(P)$ is given by:

$$\frac{d\langle \mathrm{var}(P)\rangle}{dt} = \left\langle \sum_{\text{events}} \text{rate of event} \times \text{change to } P^2 \right\rangle$$
$$-2\langle P\rangle \left\langle \sum_{\text{events}} \text{rate of event} \times \text{change to } P \right\rangle \quad (8)$$

Very similar equations can be stated for the mean number of protein molecules and the covariance between the number of mRNA and protein molecules. Putting in the possible events, we can derive:

$$\begin{aligned} \frac{d\langle P\rangle}{dt} &= \langle k_p M(+1) + \gamma_p P(-1)\rangle \\ &= k_p\langle M\rangle - \gamma_p\langle P\rangle \end{aligned} \quad (9)$$

The mean of the invariant distribution of P, denoted $\hat{P}$, is given when $d\langle P\rangle/dt = 0$ and so

$$k_p\hat{M} = \gamma_p\hat{P} \quad (10)$$

and so

$$\hat{P} = \frac{k_m k_p}{\gamma_m \gamma_p} \quad (11)$$

This is, of course, the steady state solution for the ODE version of this model. The equation for the variance of P is given by:

$$\begin{aligned} \frac{d}{dt}(\langle P^2\rangle - \langle P\rangle^2) &= \frac{d\langle P^2\rangle}{dt} - 2\langle P\rangle\frac{d\langle P\rangle}{dt} \\ &= \langle k_p M(2P+1) + \gamma_p P(-2P+1)\rangle - 2\langle P\rangle k_p\langle M\rangle + 2\langle P\rangle^2\gamma_p \\ &= k_p\langle M\rangle + \gamma_p\langle P\rangle + 2k_p\langle MP\rangle - 2k_p\langle M\rangle\langle P\rangle - 2\gamma_p\langle P^2\rangle + 2\gamma_p\langle P\rangle^2 \\ &= k_p\langle M\rangle + \gamma_p\langle P\rangle - 2\gamma_p\mathrm{var}(P) + 2k_p\mathrm{cov}(M,P) \end{aligned} \quad (12)$$

The appearance of a covariance term in this equation necessitates the derivation of a covariance equation:

$$\begin{aligned} \frac{d}{dt}(\langle MP\rangle - \langle M\rangle\langle P\rangle) &= \frac{d\langle MP\rangle}{dt} - \langle M\rangle\frac{d\langle P\rangle}{dt} - \langle P\rangle\frac{d\langle M\rangle}{dt} \\ &= \langle k_m P + \gamma_m M(-P) + k_p M^2 + \gamma_p P(-M)\rangle \\ &\quad -\langle M\rangle(k_p\langle M\rangle - \gamma_p\langle P\rangle) - \langle P\rangle(k_m - \gamma_m\langle M\rangle) \\ &= k_p\langle P\rangle - \gamma_m\langle MP\rangle + k_p\langle M^2\rangle - \gamma_p\langle MP\rangle \\ &\quad -k_p\langle M\rangle^2 + \gamma_p\langle M\rangle\langle P\rangle - k_m\langle P\rangle + \gamma_m\langle M\rangle\langle P\rangle \\ &= k_p\mathrm{var}(M) - (\gamma_m + \gamma_p)\mathrm{cov}(M,P) \end{aligned} \quad (13)$$

The values of var(M) and cov(M, P) associated with the invariant distributions are found when these two differential equations are both 0. Starting with the covariance equation, and knowing that var$(M) = k_m/\gamma_m$, we get

$$\mathrm{cov}(M, P) = \frac{k_p \mathrm{var}(M)}{\gamma_m + \gamma_p} = \frac{k_m k_p}{\gamma_m(\gamma_m + \gamma_p)} \tag{14}$$

By substituting this into the equation for the variance of P, we obtain:

$$\begin{aligned} \mathrm{var}(P) &= \frac{k_m k_p}{\gamma_m \gamma_p}\left(1 + \frac{k_p}{\gamma_m + \gamma_p}\right) \\ &= \hat{P}\left(1 + \frac{k_p}{\gamma_m + \gamma_p}\right) \end{aligned} \tag{15}$$

Mean and Variance of Protein in Negatively Regulated System

The negatively regulated system is described by the set of reactions:

- $D \mapsto D - 1;\ P \mapsto P - 1$ at rate $k_{on}DP$
- $D \mapsto D + 1;\ P \mapsto P + 1$ at rate $k_{off}(1 - D)$
- $M \mapsto M + 1$ at rate $k_m D$
- $M \mapsto M - 1$ at rate $\gamma_m M$
- $P \mapsto P + 1$ at rate $k_p M$
- $P \mapsto P - 1$ at rate $\gamma_p P$

This system is nonlinear and not time reversible. Therefore the time reversal equations cannot be used, and attempts to solve full the system using moment equations have also not been successful. Instead, we make a number of approximations which allow us to derive approximate solutions that are valid in different situations.
First, we must assume that the dynamics at the operator site are much faster than the dynamics of mRNA and protein synthesis and degradation. If this is the case, then we can consider a quasi steady state level for D, given by:

$$D = \frac{1}{1 + P/k_d} \tag{16}$$

where k_d is defined in the usual way as k_{off}/k_{on}. By looking at the consequent ODE model, we can see two behaviours of the system. The ODE model is:

$$\frac{dM}{dt} = \frac{k_m}{1 + P/k_d} - \gamma_m M \quad (17)$$

$$\frac{dP}{dt} = k_p M - \gamma_p P \quad (18)$$

To compute the steady states, we set these equations to 0 and by substituting $\hat{P} = k_p \hat{M}/\gamma_p$ we get get a quadratic equation for $\hat{M}$

$$\frac{k_p \gamma_m}{\gamma_p k_d} \hat{M}^2 + \gamma_m \hat{M} - k_m = 0 \quad (19)$$

with solution:

$$\hat{M} = \frac{-1 + \sqrt{1 + \frac{4 k_m k_p}{\gamma_m \gamma_p k_d}}}{\frac{2 k_p}{\gamma_p k_d}} \quad (20)$$

This equation leads to two natural approximations. Recall that the steady state of protein in the unregulated model, $\hat{P}_{un}$ is

$$\hat{P}_{un} = k_m k_p / \gamma_m \gamma_p. \quad (21)$$

By looking at the term inside the square root of equation 20, we see that there are two cases to be considered:

$$(i) \qquad \hat{P}_{un} \ll k_d/4 \quad (22)$$

$$(ii) \qquad \hat{P}_{un} \gg k_d/4 \quad (23)$$

In case (i), we apply the Taylor expansion $\sqrt{1+x} \simeq 1 + x/2.$ to obtain

$$\hat{M} \simeq \frac{-1 + (1 + \frac{2 k_m k_p}{\gamma_m \gamma_p k_d})}{\frac{2 k_p}{\gamma_p k_d}} = \frac{k_m}{\gamma_m} \quad (24)$$

This is the same solution as the solution of the unregulated system. Similarly $\hat{P}$ will also be the same as the unregulated system. We can think of this case as the operator being so weak compared with steady state protein level that the effect of the repression is negligible and the system behaves just like the unregulated system. Note also that the linearization is equivalent to simply dropping the quadratic term from the quadratic equation.
In case (ii), we will also apply the Taylor expansion on $\sqrt{1+x}$, but this time we must take a factor out of the square root:

$$\begin{aligned}\hat{M} &= \frac{\gamma_p k_d}{2k_p}\left(-1+\sqrt{\frac{4k_m k_p}{\gamma_m\gamma_p k_d}}\sqrt{\frac{\gamma_m\gamma_p k_d}{4k_m k_p}+1}\right) \\ &\simeq \frac{\gamma_p k_d}{2k_p}\left(-1+\sqrt{\frac{4k_m k_p}{\gamma_m\gamma_p k_d}}\left(1+\frac{\gamma_m\gamma_p k_d}{8k_m k_p}\right)\right)\end{aligned} \tag{25}$$

and by twice making use of the fact that $\hat{P}_{un} \gg k_d/4$, we obtain

$$\hat{M} = \sqrt{\frac{k_m\gamma_p k_d}{\gamma_m k_p}} \tag{26}$$

$$\hat{P} = \sqrt{\frac{k_m k_p k_d}{\gamma_m\gamma_p}} \tag{27}$$

This, therefore, gives a very useful approximation for the steady state levels of mRNA and protein for strong repressors and abundant proteins. Note that this second linearization is equivalent to dropping the $\hat{M}$ term from the quadratic equation for $\hat{M}$.

Derivation of Protein Variance

In order to derive the variance of protein abundance, we must make two further approximations. We start with the rate of protein production, which, when the repressor dynamics are fast, is given by:

$$\frac{k_m}{1+P/k_d} \tag{28}$$

Since we are interested in the case where $P \gg k_d$, we can write:

$$\frac{k_m}{1+P/k_d} \simeq \frac{k_m k_d}{P} \tag{29}$$

Observe that this is a slightly stronger approximation than that of Thattai and Van Oudenaarden who do not "cross out" the "1" from the denominator. Next, we assume that the stochastic fluctuations in P about its steady state $\hat{P}$ are small relative to the steady state, i.e. $P/\hat{P} \ll 1$. We write

$$P = \hat{P}(1 + \frac{P}{\hat{P}} - 1) \tag{30}$$

and substitute for P in Equation 29. Since $P/\hat{P} - 1 \ll 1$, we can use the Taylor series linearization for $(1+x)^{-1} \simeq 1 - x$ to obtain:

$$\begin{aligned}
\frac{k_m}{1+P/k_d} &\simeq \frac{k_m k_d}{P} \\
&= \frac{k_m k_d}{\hat{P}(1+P/\hat{P}-1)} \\
&\simeq \frac{k_m k_d}{\hat{P}}\left(2-\frac{P}{\hat{P}}\right)
\end{aligned} \tag{31}$$

This gives us a linearization of the Markov chain, with the four possible events:

- $M \mapsto M+1$ at rate $\frac{k_m k_d}{\hat{P}}\left(2-\frac{P}{\hat{P}}\right)$
- $M \mapsto M-1$ at rate $\gamma_m M$
- $P \mapsto P+1$ at rate $k_p M$
- $P \mapsto P-1$ at rate $\gamma_p P$

We can now derive equations for the variance of mRNA (denoted V_m), the variance of protein (V_p) and the covariance between mRNA and protein (C).

$$\begin{aligned}
\frac{dV_m}{dt} &= \left\langle \frac{k_m k_d}{\hat{P}}(2-\frac{P}{\hat{P}})(2M+1)+\gamma_m M(-2M+1)\right\rangle \\
&\quad -2\langle M\rangle \left\langle \frac{k_m k_d}{\hat{P}}(2-\frac{P}{\hat{P}})-\gamma_m M\right\rangle
\end{aligned} \tag{32}$$

By setting this derivative to zero, expanding the brackets, and collecting and canceling terms where relevant, we obtain:

$$\hat{P}-\frac{k_p}{\gamma_p}V_m - C = 0 \tag{33}$$

In a similar way, the equation for V_p is

$$\frac{dV_p}{dt} = \langle k_p M(2P+1)+\gamma_p P(-2P+1)\rangle - 2\langle P\rangle\langle k_p M-\gamma_p P\rangle \tag{34}$$

which leads to the steady state equation:

$$\hat{P}-V_p+\frac{k_p}{\gamma_p}C = 0 \tag{35}$$

Finally, the equation for the covariance is:

$$\frac{dC}{dt} = \left\langle \frac{k_m k_d}{\hat{P}}(2-\frac{P}{\hat{P}})P + \gamma_m M(-P) + k_p M(M) + \gamma_p P(-M) \right\rangle$$
$$- \langle M \rangle \langle k_p M - \gamma_p P \rangle - \langle P \rangle \left\langle \frac{k_m k_d}{\hat{P}}(2-\frac{P}{\hat{P}}) - \gamma_m M \right\rangle \quad (36)$$

which leads to the steady state equation:

$$C = \frac{k_p^2 V_m - \gamma_m \gamma_p V_p}{k_p(\gamma_p + \gamma_m)} \quad (37)$$

This equation for C can be substituted into equations 33 and 35 to obtain simultaneous equations for V_m and V_p. Solutions of these equations leads to the variance of protein, V_p, given by:

$$V_p = \hat{P}\left(1 + \frac{k_p - \gamma_m}{2(\gamma_p + \gamma_m)}\right) \quad (38)$$

A similar process can be followed for a system that includes a Hill Coefficient n, so that the rate of mRNA synthesis is given by:

$$\frac{k_m}{1 + (P/k_d)^n} \quad (39)$$

In this case, the variance of protein abundance is given by:

$$V_p = \hat{P}\left(1 + \frac{k_p - n\gamma_m}{(n+1)(\gamma_p + \gamma_m)}\right) \quad (40)$$

Stochastic Simulations

In support of the stochastic simulations presented in the manuscript, we ran stochastic simulations of the negative self-regulating system across a wide range of parameters in order to assess protein variability. The ranges of parameters we used were:

- k_{off}: this was varied from between $10^{-4}s^{-1}$ and $1s^{-1}$. Combined with the diffusion limited k_{on} of 0.01 per molecule per second, this means we tested k_d between $0.01nM$ and $100nM$. The steps were geometrical of size $\log_{10}(0.2)$, so that 26 different values of k_d were tested.

- k_m: this was varied between 0.01 molecules per cell s^{-1} and 1 molecule per cell s^{-1}, corresponding to mRNA synthesis times of between 1 and 100s. A geometrical step size of $\log_{10}(0.5)$ was used for this and the subsequent parameters.

- k_p: this was varied between 0.01molecules per cell s^{-1} and 1 molecules per cell s^{-1}.

- γ_m: this was varied between $0.001s^{-1}$ and $0.1s^{-1}$, corresponding to average mRNA stability between $10s$ and 17 minutes.
- γ_p: this was varied between $0.0001s^{-1}$ and $0.01s^{-1}$, corresponding to average protein stabilities of between $100s$ and 2.8 hours.

The simulations were run for 100,000 protein production and degradation events each. The results are subject to some stochasticity — particularly with weak (i.e. high) values of k_d, where the simulations are stiff — which would be improved with longer simulations, but which were not possible on the computers available to us.

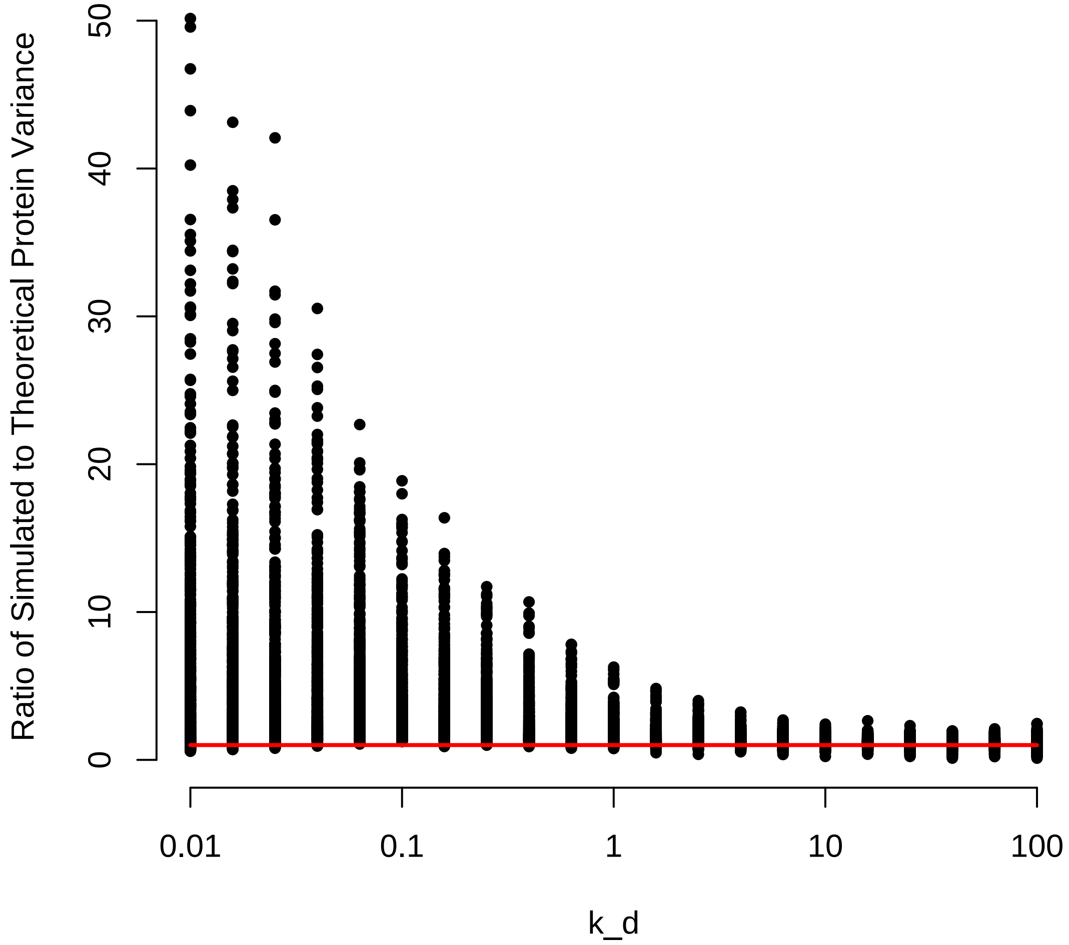

Figure 1:

The results of the simulations are summarized in Supplementary Figure 1. This figure shows the ratio between observed protein variability and theoretically predicted protein variability. A value of 1 represented by the red line is precise agreement between the two. For weak repressors ($k_d \gg 1nM$), there is broad agreement between theory and simulations — with some stochasticity. For strong repressors ($k_d \ll 1nM$), the ratio increases for many parameter ranges, with variabilities typically 10 times and as much as 50 times higher than predicted by theory.
We conclude, therefore, that the analytic approximation is good for weak repressors, and is not good for strong repressors. Further mathematical work will be needed to determine the relationship between protein variability and the model parameters for strong repressors.

In Silico Evolution

Simulations were run twenty times for each of the three scenarios. In the main text we report the best (most optimal) parameters for each of the three scenarios: minimizing protein standard deviation, rise time and mRNA abundance. Supplementary Figure 2 shows box plots for these objectives that summarize all 120 evolutionary simulations. The same overall pattern of results can be seen from all the data. The negative regulator can provide a very small improvement in controlling protein noise; a modest improvement in controlling response time and a very substantial improvement in controlling mRNA usage.
Supplementary Figure 3 shows box plots of the k_d values for the negative repressors evolved in each of the scenarios. In all cases, it can be seen that wide ranging k_d values are evolved; the same is true for other parameters (not included). The majority of noise minimization repressors are weak with k_d values between $20nM$ and $200nM$; the strongest repressor is $25nM$. The k_d values for repressors that minimize response time to 50% steady state level are also weak, the majority being from $10nM$ to $100nM$, with the strongest repressor being $5.4nM$. The k_d values for repressors that minimize response time to 100% steady state level are stronger, the majority being from $0.2nM$ to $10nM$, with the strongest repressor being $0.03nM$. Repressors that minimize mRNA levels also have strong values of k_d, the majority with values between $1nM$ and $20nM$, and the strongest repressor being $0.05nM$.

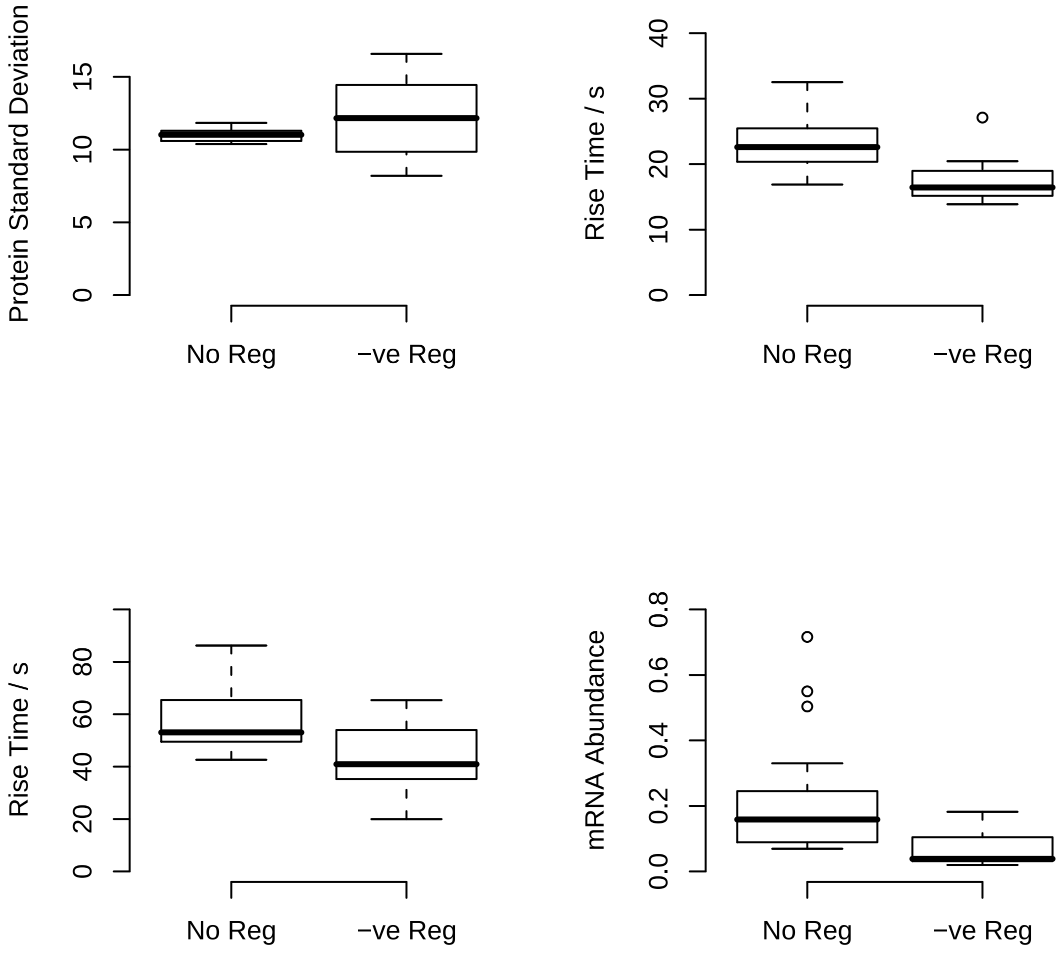
Protein Standard Deviation
0
5
10
15
No Reg
−ve Reg
Rise Time / s
0
10
20
30
40
No Reg
−ve Reg
Rise Time / s
0
20
40
60
80
No Reg
−ve Reg
mRNA Abundance
0.0
0.2
0.4
0.6
0.8
No Reg
−ve Reg

Figure 2:

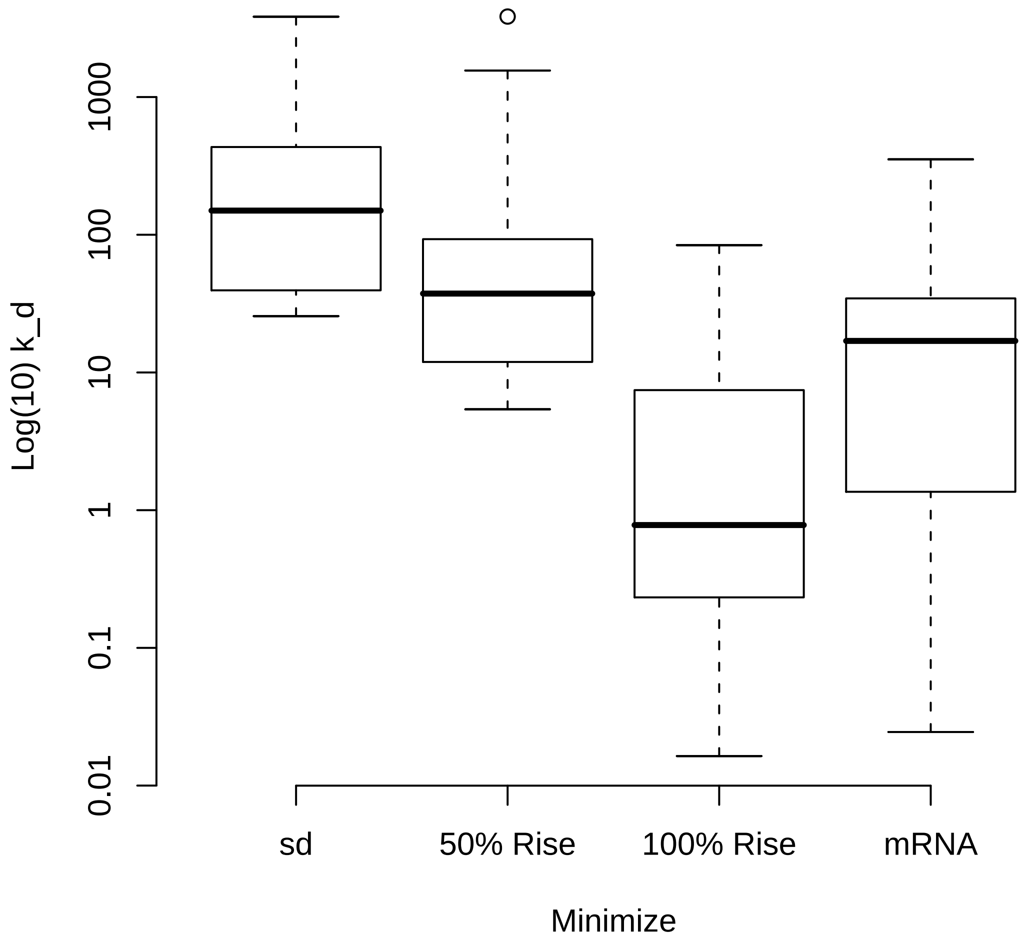

Figure 3:
